# Ileostomy 101: Understanding the Basics for Optimal Patient Care

**DOI:** 10.7759/cureus.46822

**Published:** 2023-10-11

**Authors:** Reda H Mithany, M Hasaan Shahid, Ra'ana Shahid, Abdul Hannan, Muhammad Umar Gill, Samana Aslam

**Affiliations:** 1 Laparoscopic Colorectal Surgery, Kingston Hospital NHS Foundation Trust, Kingston Upon Thames, GBR; 2 Surgery, Postgraduate Medical Institute, Lahore, PAK; 3 General Surgery, Lahore General Hospital, Lahore, PAK; 4 Surgery, Glangwili General Hospital, Carmarthen, GBR; 5 Accident and Emergency Medicine, Kings College Hospital NHS Foundation Trust, London, GBR; 6 Obstetrics and Gynaecology, Lahore General Hospital, Lahore, PAK

**Keywords:** advancements, complications with stoma, postoperative care of ileostomy, surgical techniques, ileostomy

## Abstract

This comprehensive literature review explores the foundational aspects of ileostomy, encompassing surgical techniques, postoperative care, complications, and advancements. Ileostomy, a surgical procedure redirecting the ileal lumen through an abdominal opening, is a critical intervention for various gastrointestinal conditions. The review delves into surgical techniques, emphasizing the importance of stoma location and type selection, whether temporary or permanent. Complications associated with ileostomy are discussed, highlighting the significance of vigilant postoperative care, including stoma care and addressing potential complications. The profound impact of ileostomy on patients' quality of life is elucidated, underlining the necessity for a holistic approach to patient care. Additionally, advancements in the field, such as biodegradable stoma bags, smart stoma appliances, and telemedicine, are explored for their potential to enhance patient outcomes. The review emphasizes the need for individualized approaches and ongoing research to maximize the benefits of these advancements for ileostomy patients and improve their overall experience.

## Introduction and background

Ileostomy, a surgical procedure involving the creation of an artificial opening in the abdominal wall to redirect the ileal lumen, plays a pivotal role in the management of various gastrointestinal conditions. The prevalence of ileostomies in the United States remains substantial, estimating approximately 165,000 to 265,000 individuals living with an ileostomy at any given time, and around 40,000 new ileostomies performed annually [1]. An ileostomy can be either temporary or permanent, encompassing both end and loop configurations, typically situated on the right side of the abdomen. Its primary objective is to reroute stool evacuation through the ileum, deviating from the natural pathway via the anus [2]. The genesis of surgical ileostomy dates back to 1879 when German surgeon Baum pioneered its creation to alleviate an obstructing carcinoma of the right colon. Initially, flush ileostomies were established, resulting in severe peristomal skin excoriation due to inadequate pouching systems. Over time, advancements, notably the introduction of the spouted ileostomy in 1912 by John Y. Brown and the refined technique by Dr. Bryan Brooke in 1952, revolutionized ileostomy surgery, setting the gold standard in modern colorectal surgery [3].

This literature review seeks to comprehensively elucidate the foundational aspects of ileostomy, focusing on surgical techniques, postoperative care, and their profound implications for patients' overall well-being and quality of life. The exploration delves into the surgical procedures involved in ileostomy creation, encompassing variations and advancements in techniques. Furthermore, essential aspects of postoperative care are examined, emphasizing stoma management, dietary adjustments, and lifestyle modifications. Ultimately, this review adopts a patient-centric perspective, aiming to enhance the understanding of the intricacies of ileostomy care and thereby promote improved patient outcomes and an enhanced quality of life.

## Review

Surgical techniques for ileostomy

The creation of an optimal end ileostomy or colostomy necessitates adherence to fundamental principles. These encompass the excision of a circular skin disc approximately 2.5 cm in diameter at the pre-marked site, avoiding the excision or removal of subcutaneous tissues. Subsequently, a vertical incision of approximately 3 cm in length is made in the anterior rectus sheath, with a perpendicular 1-cm incision laterally at the midpoint to guide the stoma opening away from the midline incision. The rectus abdominis muscle is split in the direction of its fibers, followed by a vertical incision in the posterior rectus sheath. The bowel is carefully delivered through the abdominal wall without any twisting, ensuring its viability.

In the context of the construction of a colostomy, it is recommended that the colon extends around 2 cm above the surface of the skin. Conversely, in the case of an ileostomy, around 5 cm of the bowel is drawn through, resulting in a fully developed ileostomy that protrudes 2 to 2.5 cm above the skin. It is worth mentioning that in order to achieve the best results, the process of eversion is required for ileostomies. The process of achieving ileostomal eversion entails the utilization of "triplicate" sutures. This technique comprises the initial suture passing through the dermis, followed by a seromuscular bite approximately 4 to 5 cm away from the proximal to the distal end of the ileum. The procedure concludes with a full-thickness bite through the cut end of the intestine. The utilization of a triple suture technique, which is oriented in a direction opposite to the mesentery, enables the successful eversion of a stoma. Standard sutures can be used to seal any remaining gaps between the triple sutures [4].

Temporary versus permanent ileostomy

The establishment of an ileostomy encompasses the potential for both temporary and permanent forms, including end or loop configurations. Temporary ileostomies find application in exigencies such as trauma or hollow viscus perforations, or as a protective measure in planned multi-step surgical interventions, preventing fecal content from reaching a distal bowel segment and mitigating anastomotic complications. Conversely, permanent ileostomies are instituted when the anorectum is excised, a scenario witnessed in cancer patients, those grappling with inflammatory bowel diseases, or instances where anastomosis is infeasible, notably due to trauma- or radiation-induced complications [5]. 

**Figure 1:**
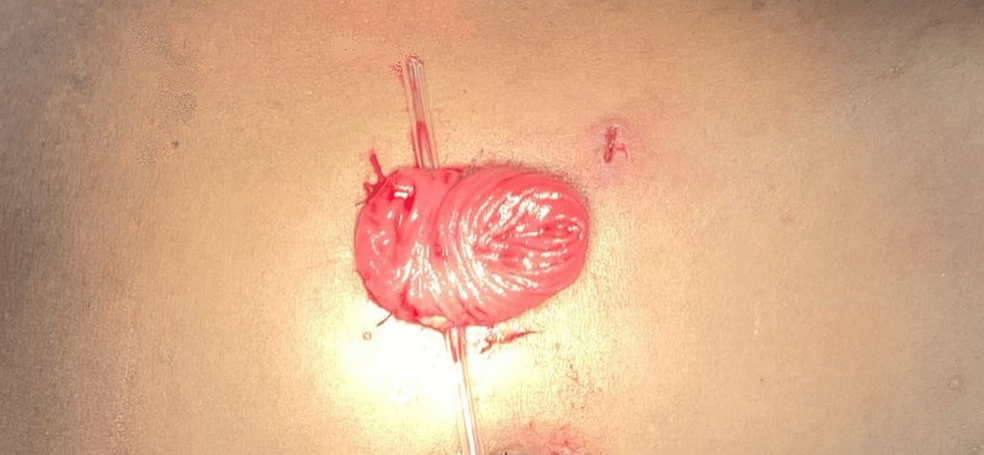
Loop Ileostomy The stoma exhibiting two discernible lumens, characterized by an intact posterior wall, thus categorizing it as a loop ileostomy. The patient depicted in the picture belongs to one of the authors, and formal consent was obtained before capturing the image.

**Figure 2:**
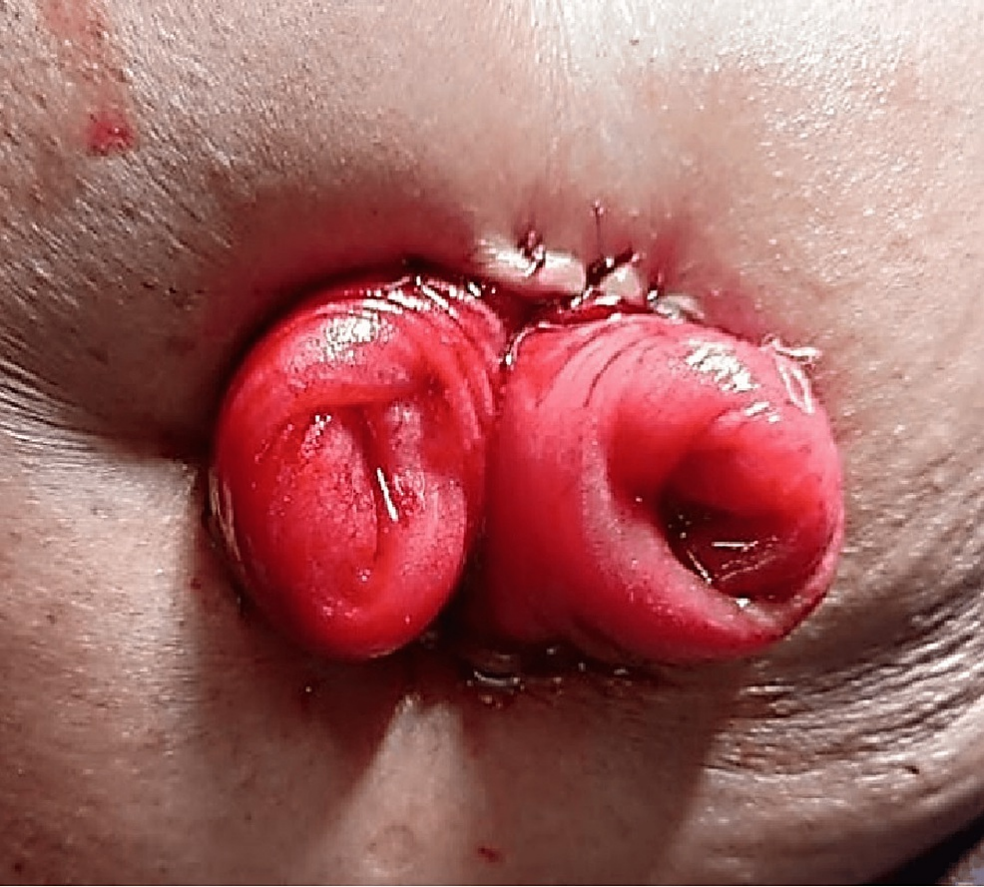
Double Barrel Ileostomy The stoma exhibiting two discernible lumens, with posterior wall not intact, thus categorizing it as a double barrel ileostomy. The patient depicted in the picture belongs to one of the authors, and formal consent was obtained before capturing the image.

**Figure 3:**
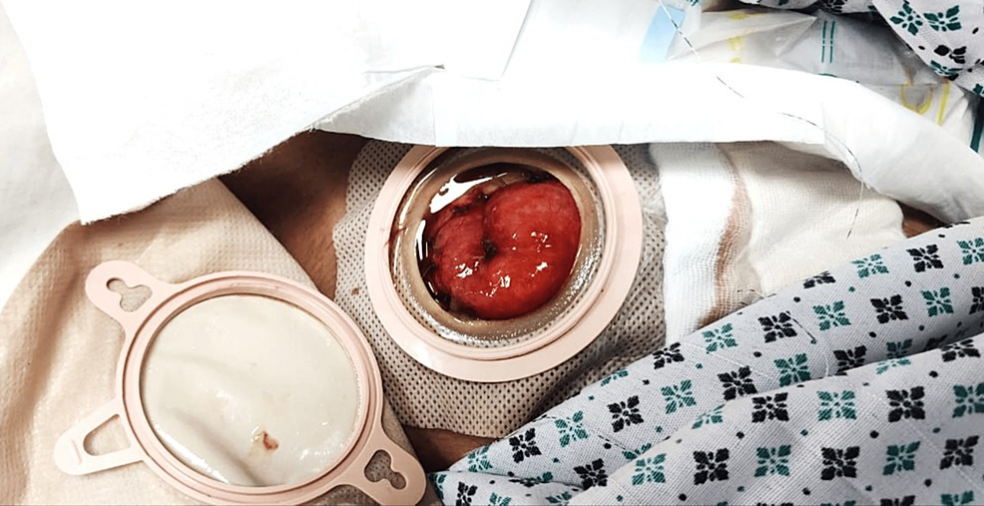
End (Terminal) Ileostomy The stoma exhibits only one lumen, thus categorizing it as an end or terminal ileostomy. The patient in the picture belongs to one of the authors, and formal consent was obtained before capturing the image.

The typology of ileostomies can be delineated into five distinct categories, contingent upon the underlying rationale driving their surgical inception. These categories encompass loop ileostomies (Figure 1), double barrel ileostomies (Figure 2), end ileostomies (Figure 3), diversion ileostomies, and defunctioning ileostomies [6-9].

Loop ileostomies involve the protrusion of an intestinal loop through an abdominal transparietal circular opening at the right iliac fossa level, subsequently secured with four interrupted sutures to avert postoperative prolapse [6]. End (terminal) ileostomies entail the division of the bowel and extraction of the proximal stump [7]. Defunctioning ileostomy, a surgical measure in colorectal surgery, serves to divert fecal flow, significantly mitigating the risk of anastomotic leakage, a critical postoperative complication [6].

Complications and challenges associated with ileostomy

**Figure 4:**
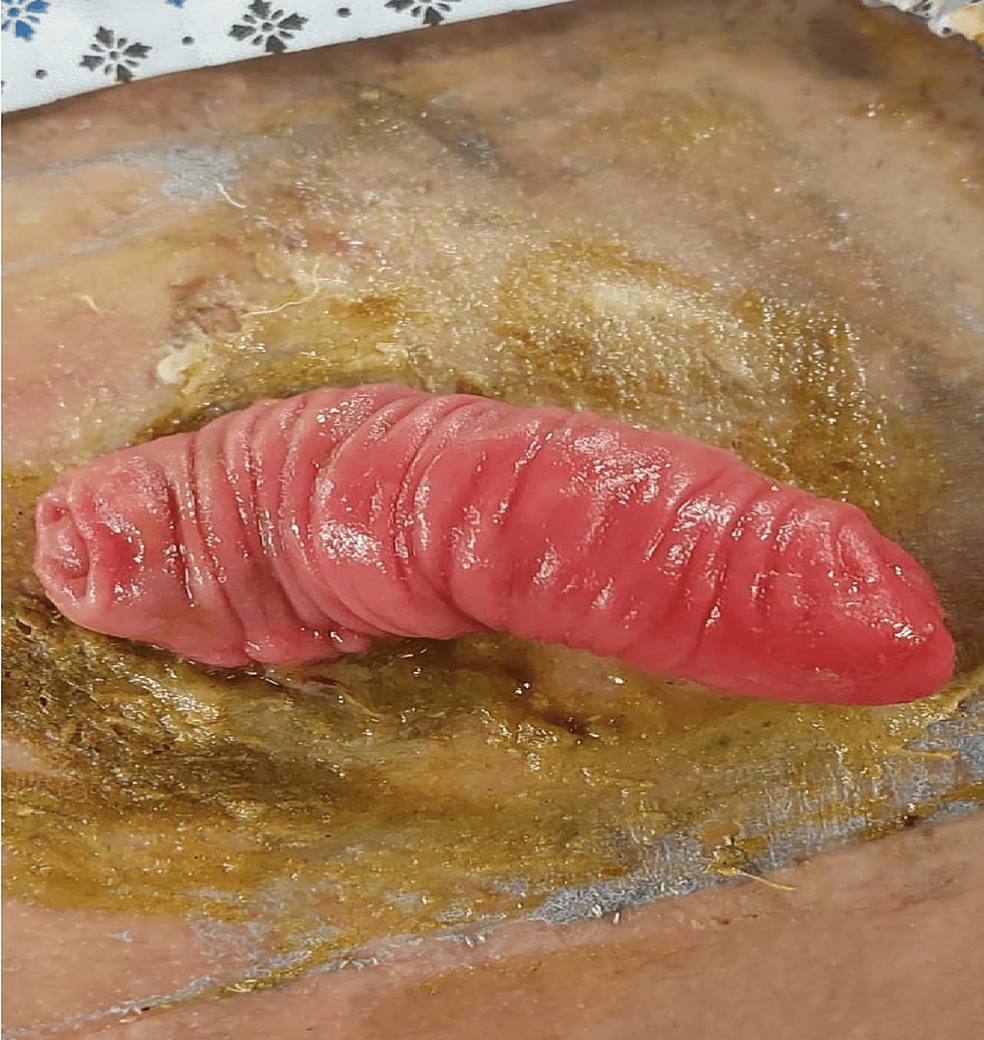
Prolapsed Ileostomy with Skin Contamination and Excoriation A visual representation of a prolapsed loop ileostomy with skin contamination and excoriation is depicted. In this instance, the protruding end of the ileostomy was not adequately enclosed within the stoma appliance or bag, resulting in contact with the surrounding skin leading to irritation and peristomal dermatitis. The skin around the prolapsed ileostomy is displaying signs of excoriation, indicating damage or abrasion due to extended contact with stool, underscoring the importance of proper management and care in such cases. The patient depicted in the picture belongs to one of the authors, and formal consent was obtained before capturing the image.

**Figure 5:**
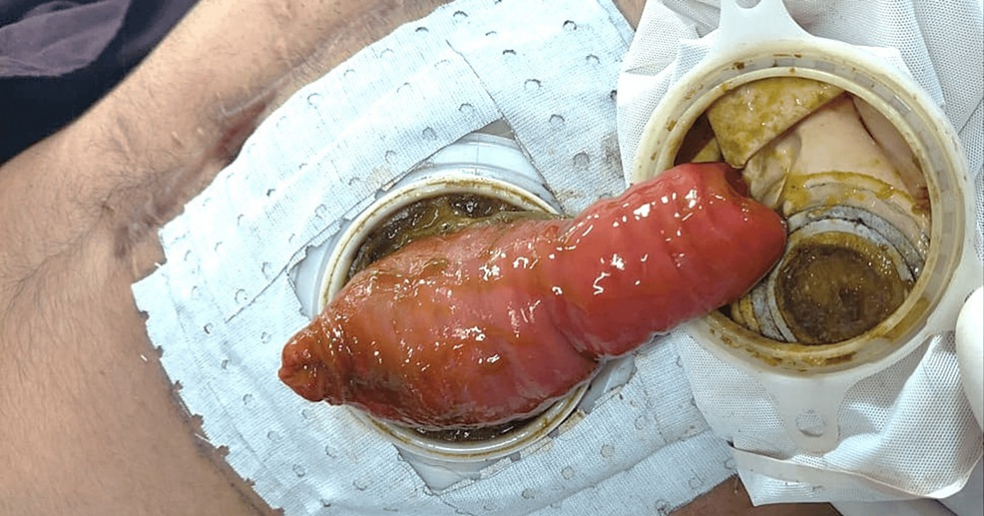
Prolapsed Ileostomy A clear depiction of a prolapsed loop ileostomy is presented. This protrusion can manifest as a noticeable bulge or projection from the abdomen, illustrating the displacement of the ileostomy. The patient in the picture belongs to one of the authors, and formal consent was obtained before capturing the image.

Ileostomy presents a viable approach to curbing morbidity and mortality linked with anastomotic leakage in both colonic and small gut anastomoses. Furthermore, ileostomies prove instrumental in reducing morbidity in septicemic patients afflicted by ileal perforation stemming from conditions like typhoid fever, tuberculosis, trauma, or ruptured appendix. However, complications, including dehydration due to heightened ileostomy output leading to fluid and electrolyte loss, manifest in up to 16.9% of cases within the initial 60 days. Additional complications encompass skin excoriation (Figure 4), wound infection, nonfunctioning stoma, prolapse (Figure 5), stenosis, retraction, high output fistula, parastomal hernia, necrosis, and hemorrhage [10].

Postoperative care and management

The task of altering a stoma bag can pose a notable challenge for the patient. A methodical approach is presented herein to facilitate a proficient bag-changing process. Initial steps involve rigorous hand hygiene. Subsequently, essential equipment, such as a small bowl of water, wipes for peristomal skin cleansing and drying, a new appliance, a stoma-measuring device, and a disposal bag, are assembled. For drainable appliances, appropriate emptying into a receptacle is advised. Gentle removal and disposal of the soiled appliance, coupled with thorough cleansing of the peristomal skin, constitute crucial steps. Precise stoma measurement ensures a well-fitting new appliance, further securing it without constriction. A meticulous conclusion involves discarding used materials and a final round of hand hygiene [11].

Stoma Care

Best practices and importance in preventing complications: An integral facet of optimizing ileostomy outcomes entails commencing patient education and preparation for ostomy adaptation during the preoperative period. Active involvement in ostomy support groups and guidance from certified wound ostomy continence nurses significantly reduce complication rates and improve long-term outcomes and psychosocial adjustment. Preoperative stoma site marking, orchestrated by enterostomal therapists or seasoned surgeons, emerges as a pivotal measure, notably curbing postoperative stoma-related complications [12]. Addressing peristomal skin complications necessitates an adaptive pouching system, topical therapy for healing, and maintaining a robust seal between the pouch appliance and the skin. Prudent management, supported by vigilant follow-up and collaborative care, is essential in addressing challenges and ensuring optimal outcomes in ostomy care [13].

Ischemia, arising from inadequate blood supply to the exposed bowel section, results in mucosal sloughing and potential venous congestion. Prolonged venous congestion may compromise arterial blood supply, leading to stoma necrosis. Preparing the intended bowel limb for the stoma well in advance of the operation can help recognize and mitigate ischemia by allowing demarcation and potential further resection if needed. However, this approach is not foolproof, and inadvertent ischemia can occur during stoma creation, particularly by dividing excessive mesentery to achieve a tension-free stoma, especially in obese patients. Immediate post-operative ischemia may also result from bowel edema, which can lead to subsequent mucosal healing but could be followed by mucocutaneous separation and stenosis. In very severe cases of retraction, the stoma retracts and separates from the skin. Risk factors for mucocutaneous separation include malnourishment, ischemia, tension, and poor suturing. In severe cases, emergency surgery and revision are required, as peritonitis may result [14].

A parastomal hernia, occurring in nearly 78% of stoma patients within the initial two years after surgery but potentially up to 20 to 30 years post-surgery, is a type of incisional hernia located near or right next to the stoma. Symptoms in affected individuals often include peristomal bulging during coughing, stoma-related discomfort or pain, and challenges in keeping the stoma appliance secure, resulting in leaks. A physical examination, resembling assessment for other incisional hernias, reveals a noticeable bulge adjacent to the stoma during a Valsalva maneuver in a standing position. Detectable fascial defects near the stoma further confirm the condition. Timely ostomy reversal can mitigate the risk for these patients. For those with milder symptoms, conservative management with well-designed stomal support suffices. However, surgical intervention becomes necessary in cases of obstruction, incarceration, or strangulation of the hernia. Surgical options such as simple fascial repair, stoma translocation, and mesh repair are available for those requiring surgical intervention [15].

Quality of life and patient perspectives

The presence of a stoma can lead to alterations in body perception and exert a substantial impact on the physiological, psychological, emotional, and interpersonal aspects of individuals. The attainment of a comprehensive treatment approach for patients necessitates the presence of a high standard of quality of life. A research study conducted in China aimed to evaluate the quality of life of individuals with stomas by utilizing a stoma self-care agency scale and health hope index. The findings of the study indicated that patients encountered challenges in their occupational and social environments. Other problems that were raised include issues related to sexuality, body image, and the stoma itself. The present study aimed to evaluate the enduring impact of ostomy surgery on the overall well-being of individuals affiliated with the United Ostomy Association of America. A comprehensive questionnaire was employed to gather data pertaining to the long-term effects experienced by these individuals after a period of five years following their surgical procedure. The report has demonstrated that patients experience improved well-being as they experience an extended lifespan with the stoma. The quality of life of Iranians has been examined through research conducted by the ostomy society. This study has identified various factors that impact their quality of life, including the specific type of ostomy procedure, the underlying disease that necessitated the formation of the stoma, post-ostomy depression, dissatisfaction with sexual activities, challenges related to the placement of the ostomy, and alterations in clothing preferences [16].

The enduring presence of an ostomy can be a distressing encounter that imposes substantial limitations on an individual's personal and social spheres, so greatly impacting their psychological and social welfare. According to contemporary research, individuals with ostomies frequently encounter adverse emotional states, including but not limited to wrath, anxiety, and dread. The substantial impact of an ostomy on a patient's daily routine encompasses all elements of life, including romantic relationships, professional conditions, and financial circumstances, leading to potential unfavorable effects. The process of adapting to this novel reality necessitates efficient communication, acceptance, and comprehension, which are all long-term endeavors. The continuous endeavors of patients to improve their quality of life are persistent, and they might derive advantages from emotional and organizational assistance, autonomy, education, and self-management. Nurses assume a crucial position in providing assistance to ostomy patients, facilitating their empowerment, delivering support, cultivating acceptance, and aiding in the cultivation of good coping mechanisms to manage and adjust to their altered life circumstances [17].

Advancements in the field of ileostomy

Biodegradable Stoma Bags 

Traditional stoma bags are made of non-biodegradable materials, which can contribute to environmental waste. Biodegradable stoma bags were being developed to reduce the ecological impact of ileostomy care.

Smart Stoma Appliances 

Innovative stoma appliances with embedded sensors were being developed to monitor output levels, temperature, and pH. This data could be transmitted to healthcare providers to help in the early detection of potential complications [18].

Bacterial Therapies 

Researchers have investigated the use of beneficial bacteria (probiotics) to promote a healthier gut microbiome and potentially reduce complications like peristomal skin issues [19].

Telemedicine and Remote Monitoring

Telemedicine and remote monitoring solutions were increasingly being used to support ileostomy patients. These technologies allow healthcare providers to monitor stoma function and provide timely guidance and support to patients without requiring them to visit the hospital frequently [20].

3D Printing 

3D printing technology was being explored for customizing stoma appliances to fit patients' specific anatomical needs, improving comfort, and reducing leaks or skin irritation [21].

Patient Education and Support Apps 

Mobile apps and digital platforms were being designed to provide educational resources, lifestyle tips, and peer support for ileostomy patients, helping them manage their condition more effectively [22].

It's important to note that while these emerging technologies and interventions show promise in improving ileostomy outcomes, their effectiveness may vary from person to person. Furthermore, the adoption of these innovations may depend on factors like geographic location, healthcare infrastructure, and individual patient preferences.

## Conclusions

Ileostomy, an integral surgical intervention for various gastrointestinal conditions, significantly impacts patients' lives. Surgical techniques are critical in ensuring a successful ileostomy, with careful consideration of the stoma's location and type (end, loop, double barrel, etc.). Understanding the indications for temporary versus permanent ileostomies is essential for tailored patient care. Complications associated with ileostomy necessitate vigilant postoperative care and management, with an emphasis on stoma care, skin complications, and potential issues like ischaemia, retraction, and parastomal hernias.

The literature highlights the profound influence of ileostomy on patients' quality of life, emphasizing the need for a holistic approach to their care. Psychological and emotional support are crucial aspects of managing the adaptation to life with a stoma, and patient education plays a vital role. Advancements in the field, such as biodegradable stoma bags, smart stoma appliances, and telemedicine, offer promising avenues for improving patient outcomes and enhancing their overall experience. However, individualized approaches and continued research are essential to maximizing the benefits of these advancements for ileostomy patients.
